# Different vegetal protein hydrolysates distinctively alleviate salinity stress in vegetable crops: A case study on tomato and lettuce

**DOI:** 10.3389/fpls.2023.1077140

**Published:** 2023-02-15

**Authors:** Monica Yorlady Alzate Zuluaga, Sonia Monterisi, Youssef Rouphael, Giuseppe Colla, Luigi Lucini, Stefano Cesco, Youry Pii

**Affiliations:** ^1^ Faculty of Science and Technology, Free University of Bozen/Bolzano, Bolzano, Italy; ^2^ Department of Agricultural Sciences, University of Naples Federico II, Portici, Italy; ^3^ Department of Agriculture and Forest Sciences, University of Tuscia, Viterbo, Italy; ^4^ Department for Sustainable Food Process, Research Centre for Nutrigenomics and Proteomics, Università Cattolica del Sacro Cuore, Piacenza, Italy

**Keywords:** abiotic stress, antioxidant defense system, proline, salt-tolerant crop, salt-sensitive crop

## Abstract

Plants have evolved diverse plant-species specific tolerance mechanisms to cope with salt stress. However, these adaptive strategies often inefficiently mitigate the stress related to increasing salinity. In this respect, plant-based biostimulants have gained increasing popularity since they can alleviate deleterious effects of salinity. Hence, this study aimed to evaluate the sensitivity of tomato and lettuce plants grown under high salinity and the possible protective effects of four biostimulants based on vegetal protein hydrolysates. Plants were set in a 2 × 5 factorial experimental design completely randomized with two salt conditions, no salt (0 mM) and high salt (120 mM for tomato or 80 mM for lettuce), and five biostimulant treatments (C: *Malvaceae*-derived, P: *Poaceae*-derived, D: Legume-derived commercial ‘Trainer^®^’, H: Legume-derived commercial ‘Vegamin^®^’, and Control: distilled water). Our results showed that both salinity and biostimulant treatments affected the biomass accumulation in the two plant species, albeit to different extents. The salinity stress induced a higher activity of antioxidant enzymes (e.g., catalase, ascorbate peroxidase, guaiacol peroxidase and superoxide dismutase) and the overaccumulation of osmolyte proline in both lettuce and tomato plants. Interestingly, salt-stressed lettuce plants showed a higher accumulation of proline as compared to tomato plants. On the other hand, the treatment with biostimulants in salt-stressed plants caused a differential induction of enzymatic activity depending on the plant and the biostimulant considered. Overall, our results suggest that tomato plants were constitutively more tolerant to salinity than lettuce plants. As a consequence, the effectiveness of biostimulants in alleviating high salt concentrations was more evident in lettuce. Among the four biostimulants tested, P and D showed to be the most promising for the amelioration of salt stress in both the plant species, thereby suggesting their possible application in the agricultural practice.

## Introduction

It is well known that plants, being sessile organisms, must exhibit a certain adaptive plasticity to survive when unfavorable or stressful factors are present in their growing environment (Zhu, 2016). Therefore, to efficiently express the adaptive responses to specific abiotic stresses, the quick reaction to environmental changes appears to be crucial (Nguyen et al., 2016). Among these stresses, salinity is certainly one of the most devastating, causing severe damage to plant growth and productivity and threatening food security. Around 20% of the world’s cultivable lands (about 300 Mha) are impaired by high salinity, with an estimated annual global loss of 12 billion USD (Behera et al., 2022).

Vegetable crops are particularly susceptible to salinity stress compared to other agricultural crops (Machado and Serralheiro, 2017). In fact, the majority of vegetable crops have a low salinity threshold (EC_t_) that ranges from 1.0 to 2.5 dS m^−1^ in saturated soil. However, it should be noted that the severity of salinity effects is variable among different plant species (Abiala et al., 2018). For instance, onion and carrot are considered salt-sensitive vegetable crops (EC_t_ < 1.2), potato, tomato and lettuce are moderately sensitive (1.7 < EC_t_ < 2.5), while asparagus has been classified as the most salt-tolerant vegetable crop (EC_t_ > 4.0) (Machado and Serralheiro, 2017). As concerns the plant effects, salinity can negatively alter morpho-physiological and biochemical functions at extents that are plant species-specific, thus resulting in nutritional and ion imbalance, oxidative and osmotic stress, damage to the cell membranes, proteins and photosynthetic machinery, and a decrease in plant growth and yield (Hasanuzzaman and Fujita, 2022).

To face salt stress and its effects, plants have developed different adaptive mechanisms, including the production of enzymes (*e.g.*, ascorbate peroxidase - APX, catalase - CAT, superoxide dismutase - SOD, monodehydroascorbate reductase - MDHAR) and molecules (*e.g.*, ascorbic acid, phenolic compounds, alkaloids, α-tocopherols) with antioxidant activity, and compatible osmolytes (*e.g.*, proline, glycine, betaine) (Ismail et al., 2014; Zaid and Wani, 2019; Behera et al., 2022). In addition, the modulation of the levels of endogenous phytohormones (*e.g.*, auxins, abscisic acid, salicylic acid, jasmonic acid, brassinosteroids) and the downstream changes in roots, leaves and cellular structures are also important response mechanisms (Fariduddin et al., 2019; Sadiq et al., 2020; Zaid et al., 2021; Behera et al., 2022). However, these adaptive strategies might not be enough to efficiently overcome the limitations imposed by salt stress. Therefore, the acquisition of new knowledge appears crucial for the development of agronomic approaches/practices that can strengthen the adaptive response of plants to salt stress. In this respect and in a framework of increasingly sustainable agriculture, different approaches based on the use of natural products have been developed.

Among these products, the class of plant biostimulants (PBs) encompasses a wide variety of effectors, including organic or inorganic substances and/or microorganisms, and they have recently emerged as potential and eco-friendly tools to improve plant growth, productivity and alleviate the negative effects of abiotic stresses (Bulgari et al., 2019). Vegetal-derived protein hydrolysates (PHs) are a particular category of PBs, formed by a mixture of soluble peptides and free amino acids with potential bioactive effects aimed at enhancing plant growth and nutrition as well as at improving tolerance to salt stress following leaves or roots application (Colla et al., 2017). The mechanisms underlying the protective action of PHs in the salinity stress mitigation may include: i) regulation of key enzymes involved in the TCA-cycle and N-assimilation pathway (Colla et al., 2017); ii) increased photosynthetic metabolism by the elicitation of hormone-like activities (Di Mola et al., 2021); iii) modulation of the phenylpropanoids metabolism (Bavaresco et al., 2020); iv) changes in the gene expression of certain stress-inducible proteins (Vaseva et al., 2022).

Considering the potential role of PHs in mitigating the harmful effects of abiotic stresses, their use in vegetable species, which are more prone to salinity stress, represents a feasible strategy to encounter the negative impact of high salt concentrations. Among vegetable crops, tomato constitutes one of the most important fruiting vegetable crop in the world (Behera et al., 2022), whereas lettuce is one of the most consumed leafy vegetables (Shin et al., 2020). However, for both the negative effect of salt stress on the growth, biomass accumulation and yield are well described (Rouphael et al., 2017; Alam et al., 2021).

Based on the premises previously reported, and also considering the increasing global concerns about salinity as well as the economic and nutritional importance of vegetable crops, this work aims at investigating i) the different sensitivity of tomato and lettuce plants to salinity stress, ii) the constitutive biochemical mechanisms (*i.e.*, activation of antioxidant enzymes, osmolyte accumulation) underpinning the different plant response to salinity in the short-term and iii) the effects of four PHs, obtained from different vegetal sources, in eliciting protective mechanisms (*i.e.*, osmolyte accumulation, antioxidant defense system, modulation of key genes and ion homeostasis) in tomato and lettuce grown under optimal and salt-stress conditions. Considering that PHs have been described as plant species-specific and origin-specific (Parađiković et al., 2019), we hypothesize that the effects observed on one particular PH-plant species combination could not be directly generalized to other PHs or other vegetable crops. For these reasons, we adopted a fully randomized experimental design based on two plant species, two salinity levels and five different treatments (four biostimulants and a negative control), focusing our investigations on biochemical and molecular parameters in a short-term experiment.

## Materials and methods

### Vegetal-derived biostimulants

**Figure 1:**
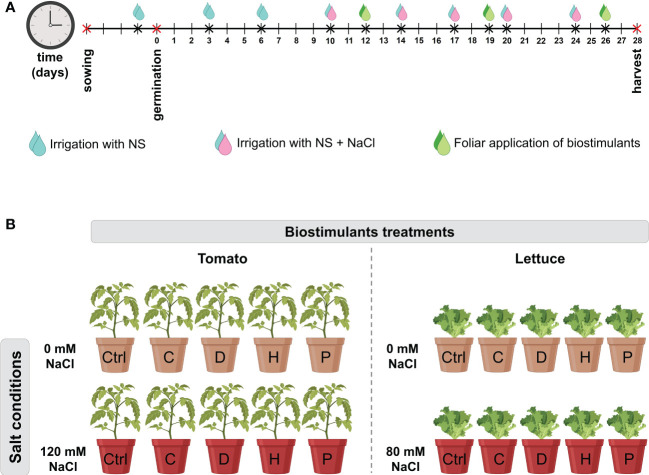
**(A)** Timeline of plant cultivation and treatments. Tomato and lettuce seeds were germinated for 5 days. After germination, seedlings were irrigated twice a week with 40 mL of NS. At 10 DAG, salinity condition was imposed by irrigating plants with NS supplemented with NaCl (120 mM for tomato and 80 mM for lettuce plants). Salt treatment was repeated twice a week. Starting from 12 DAG, biostimulants were foliarly applied once a week. Sampling was carried out at 28 DAG. **(B)** The experimental design of the present study. Plants were set in a 2 × 5 factorial experimental design completely randomized with two salt conditions: no salt (0 mM) and high salt (120 mM for tomato or 80 mM for lettuce); and four biostimulant treatments with the PHs (C, D, H and P) plus a Control (Ctrl) treatment with distilled water.

Four protein hydrolysates (PHs) plus a control (consisting only of distilled water) were used in this experiment. Two of the biostimulants were commercial products resulting from enzymatic hydrolysis of legume-derived proteins: Trainer^®^ (D) and Vegamin^®^ (H) commercialized by Hello Nature USA Inc. (Anderson, IN 46016, US). The other two were provided by the Department of Agriculture and Forest Sciences (University of Tuscia, Italy) which were obtained by enzymatic hydrolysis of *Malvaceae* (C) and *Poaceae* (P) biomass, as previously described (Ceccarelli et al., 2021; Sorrentino et al., 2021). The biostimulants were prepared at a concentration of 3 mL L^−1^ of water solution and then evaluated through foliar application. Plants were exposed to the biostimulants once a week until the harvest (**Figure 1A**).

### Plant growth conditions and experimental design

Tomato (*Solanum. lycopersicum* L. cv MicroTom) and lettuce (*Lactuca. sativa* L. cv Aquino) plants were grown in 200 mL pots filled with 150 g of a substrate obtained by mixing sand and sieved peat (Substrate 2, Klasmann-Deilmann GmbH, Germany) in a proportion of 1:1 (*w/w*) ratio. Two hours before sowing, pots were irrigated with 40 mL of distilled water and afterwards two seeds were sown at a depth of 1 cm into each pot and placed into a climatic chamber (conditions: 14/10-h light/dark period, 24/19 °C, 250 μmol m^−2^ s^−1^ light intensity, and 70% relative humidity). After germination, seedlings were thinned to one plant per pot and irrigated twice a week with 40 mL of a modified Hoagland’s solution (NS) composed as follows: 0.36 g L^-1^ Ca (NO_3_)_2_, 0.1 g L^-1^ KH_2_PO_4_, 0.80 g L^-1^ KNO_3_, 0.04 g L^-1^ NH_4_NO_3_, 0.13 g L^-1^ MgSO_4_, and 0.01 mg L^-1^ of Mikron fertilizer (Cifo Srl, Italy) (**Figure 1A**). At ten days after germination (DAG), when seedlings have reached the 2-true-leaf stage, salinity condition was imposed by irrigating plants with NS supplemented with NaCl at the final concentration of 120 mM for tomato plants or 80 mM for lettuce plants, as described by Sorrentino et al. (2022). Plants were subjected twice a week to salt application, resulting in a total of five applications throughout the whole experimental period (**Figure 1A**). Starting from 12 DAG, biostimulants were applied through foliar spraying once a week as described by (Zuluaga et al., 2022). A total of three foliar applications of PHs were done throughout the experiment (**Figure 1A**). Summarizing, plants were set in a 2 × 5 factorial experimental design completely randomized with two salt conditions: no salt (0 mM) and high salt (120 mM for tomato or 80 mM for lettuce); and four biostimulant treatments with the PHs (C, D, H and P) plus a Control treatment with distilled water (**Figure 1B**). Three biological replicates with two plants per replicate were performed for each treatment. At 28 DAG, plants were harvested: one plant of each biological was dried to a constant weight at 65°C following the determination of root dry weight (RDW) and shoot dry weight (SDW), while leaves from the remaining plant were immediately frozen in liquid nitrogen, then stored at -80°C until use.

### Sodium and potassium content

Leaf tissues dried at 65°C were ground to a fine powder using Tissue Lyser II. Approximately 0.2–0.3 g of sample was weighed and acid digested with 69% ultrapure HNO_3_ (Carlo Erba, Milano, Italy) in a single reaction chamber microwave digestion system (UltraWAVE, Milestone, Shelton, CT, USA). The digested samples were diluted to 2% HNO_3_ with ultrapure grade water (18.2 MΩ·cm at 25°C), and then the concentration of Na and K was determined using an inductively coupled plasma–mass spectrometer (ICP-MS, iCAP™ RQ, Thermo Scientific). Element quantification was carried out using certified multi-element standards (CPI International, https://cpiinternational.com). NIST standard reference materials 1573a (tomato leaves) and 1570a (spinach leaves) were used as external certified references, which were digested and analyzed the same way as the samples.

### Proline content

Free proline content was determined *via* reaction with ninhydrin according to the method described by Bates et al. (1973). Briefly, 0.5 g of leaf samples frozen in liquid nitrogen were homogenized in 10 mL of 3% sulfosalicylic acid and centrifuged at 3000 x *g* at 4°C for 10 min. Two milliliters of supernatant were reacted with 2 mL of freshly prepared acid-ninhydrin reagent for 1 h at 90°C. The reaction was then stopped by an ice bath. The chromophore was extracted using 4 mL of toluene and the absorbance at 520 nm was recorded. The proline concentration was estimated through a calibration curve and data were expressed as µg proline per g fresh weight (µg g^-1^ FW).

### Antioxidant enzyme activity

The enzymatic extract was prepared by grinding 0.5 g of frozen leaves in 5 mL of extraction buffer (100 mM potassium phosphate buffer, pH 7.5, containing 0.5 mM EDTA). The homogenate was centrifuged at 10,000 x *g* and 4°C for 10 min. The enzymatic extract was collected and subsequently used to determine APX, GPX, CAT, SOD and the total protein content determined by the Lowry method (Lowry et al., 1951) with bovine serum albumin as a standard curve.

Ascorbate peroxidase (EC 1.11.1.11) was assessed by following the consumption of ascorbate at 290 nm (Nakano and Asada, 1981). The APX activity was estimated based on the molar extinction coefficient of 2.8 mM^-1^ cm^-1^ and expressed in µmol ascorbate mg^-1^ protein min^-1^. Guaiacol peroxidase (GPX, EC 1.11.1.7) activity was estimated by measuring the formation of tetraguaiacol at 470 nm (Castillo et al., 1984). The activity of the enzyme was calculated using the molar extinction coefficient of 26.6 mM^-1^ cm^-1^ and expressed in µmol tetraguaicol mg^-1^ protein min^-1^. Catalase (CAT, EC 1.11.1.6) activity was determined by following the consumption of H_2_O_2_ at 240 nm (Aebi, 1984). The enzyme activity was calculated based on the molar extinction coefficient of 39.4 mM^-1^ cm^-1^ and expressed in µmol H_2_O_2_ mg^-1^ protein min^-1^. Superoxide dismutase (SOD, EC 1.15.1.1) activity was measured at 560 nm using the photochemical reduction of nitroblue tetrazolium, NBT (Dhindsa et al., 1981). SOD activity was expressed on protein basis as units mg^-1^ protein. All the determinations have been performed on three independent biological replicates, whereby each biological replicate was formed by a pool of two plants.

### Gene expression analysis

Leaf tissues frozen in liquid nitrogen were ground to a fine powder. Total RNA was extracted from 100 mg of ground leaves using the Spectrum Plant Total RNA Kit (Sigma- Aldrich, St. Louis, MO, USA) according to the manufacturer’s instructions. The total RNA (1 μg) was treated with 10U of DNAse RQ1 to degrade possible DNA contamination, and cDNA was synthesized using the ImProm-II Reverse Transcription System (Promega, Madison, WI, USA) following the manufacturer’s instructions. Gene-specific primers were designed for the target gene, as well as for the housekeeping gene, the elongation factor 1α (**Supplementary Table 1**). Quantitative real-time reverse transcription PCR (qRT-PCR) was carried out in triplicate with the following conditions: 5 min at 95°C, followed by 40 cycles at 95°C for 30 s and 55°C for 30 s. The housekeeping transcript was used to calculate the mean normalized expression value (MNE; (Simon, 2003)) for each sample and the relative expression ratio values were calculated by the 2^−ΔΔCt^ method according to Livak and Schmittgen (2001).

### Statistical analysis

All the experimental data for both plant species (*S. lycopersicum* and *L. sativa*) were statistically subjected to two-way ANOVA using R software (version 4.0.3). The mean values were separated according to Tukey’s HSD test with p < 0.05, and salinity levels effects were compared using the t-test. The following R packages were used for data visualization and statistical analyses: ggplot2, agricolae and ggpbur.

## Results

### Plant biomass

The biomass accumulation of both plant species was strongly influenced by salt stress. The results showed that dry matter accumulation in lettuce was more affected by salinity than in tomato plants. Moreover, the root system underwent a more pronounced decrease than the aerial parts, in both plant species. For instance, the root dry weight (RDW) of tomato under saline conditions, independently of the treatment applied, was significantly decreased by 58-65%, whilst in lettuce, the drop ranged between 47-72% compared to the no-salt condition (**Table 1**). On the other hand, the shoot dry weight (SDW) of tomato was reduced by about 28-42% under high salinity, whilst a decrement of 17-58% was observed in lettuce plants (**Table 1**). However, the application of PHs induced differential effects in each plant species, and they were dependent on the nature of the biostimulant applied and the salinity conditions. In the specific case of lettuce plants, all PHs applied stimulated the biomass accumulation in roots and shoots under high salinity compared to the saline control. Yet, biostimulant P induced the most remarkable effects enhancing RDW and SDW by more than 130%. Nonetheless, under no salt conditions, only biostimulant P induced the most significant effects in increasing lettuce biomass when compared to the untreated control (**Table 1**). Regarding tomato plants growing under salinity stress, only the application of PHs D and P significantly enhanced RDW (by 22% and 32%, respectively). At the same time, no significant effects were induced by the PHs on the SDW. However, under no-salt conditions, PHs D and P were also efficient in increasing RDW of tomato plants by about 20%. In contrast, C, H and P enhanced the accumulation of SDW by more than 15% compared to control plants (**Table 1**). In addition, as high salt concentrations inhibited root growth more than shoot growth, the expected reduction of root-to-shoot ratio (R/S) was also observed in both plant species (**Table 1**). Nevertheless, tomato plants treated with biostimulant D presented a higher R/S ratio under both salinity conditions, whilst biostimulant P was notably better for lettuce plants.

**Table 1:** Root dry weight (RDW), shoot dry weight (SDW) and root to shoot (R/S) ratio of tomato and lettuce grown under salinity stress and protein hydrolysates application.

Parameters	Salt levels	Tomato					Lettuce				
		Biostimulant treatment					Biostimulant treatment				
		Control	C	D	H	P	Control	C	D	H	P
RDW	No salt	0.099 Ab	0.110 Aab	0.119 Aa	0.106 Aab	0.120 Aa	0.047 Acd	0.040 Ad	0.055 Ab	0.051 Abc	0.073 Aa
	High salt	0.037 Bc	0.038 Bbc	0.045 Bab	0.044 Babc	0.049 Ba	0.013 Bd	0.021 Bbc	0.024 Bb	0.019 Bc	0.033 Ba
SDW	No salt	0.400 Ac	0.465 Aa	0.403 Abc	0.492 Aa	0.458 Aab	0.377 Ab	0.370 Ab	0.401 Ab	0.408 Ab	0.522 Aa
	High salt	0.287 B	0.289 B	0.266 B	0.286 B	0.291 B	0.159 Bd	0.307 Bb	0.292 Bb	0.237 Bc	0.365 Ba
R/S Ratio	No salt	0.248 Abc	0.236 Abc	0.295 Aa	0.216 Ac	0.263 Aab	0.124 Aab	0.109 Ab	0.138 Aa	0.133 Aa	0.139 Aa
	High salt	0.128 Bb	0.130 Bab	0.170 Ba	0.153 Bab	0.168 Bab	0.072 Bb	0.069 Bb	0.083 Bab	0.082 Bab	0.092 Ba

Differences between biostimulant treatments were determined using Tukey’s HSD test, and significant differences (p < 0.05) are indicated by different lowercase letters when comparing means in rows. Salt level effects were compared using Student’s t-test, and significant differences (p < 0.05) are indicated by different capital letters when comparing means in columns. No significant differences are indicated by omitting notation letters.

### Ion homeostasis

**Figure 2:**
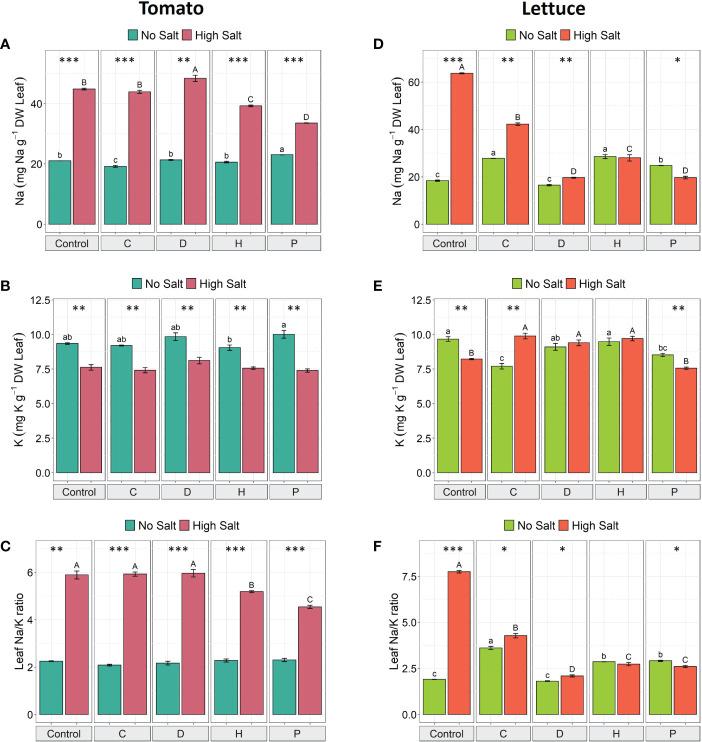
The concentration of Na^+^, K^+^ and Na^+^/K^+^ ratio in leaves of tomato **(A–C)** and lettuce **(D–F)** grown under salinity stress and protein hydrolysates application. Values are means ± SE. Lowercase letters compare treatments under no salt, and capital letters compare treatments under high salt. Equal letters correspond to average values that do not differ according to Tukey’s HSD test (p < 0.05). Asterisks indicate significant differences between high and no salt, according to Student’s *t-*test (^*^p < 0.05, ^**^p < 0.01, ^***^p < 0.001).

To investigate whether PHs application could mitigate salt stress in both plant species, we measured leaves’ Na^+^ and K^+^ content. The salt stress significantly increased Na^+^ content (ranging from 46% to 129%) and decreased K^+^ content (ranging from 16% to 26%) in tomato under all treatments (**Figures 2A, B**). However, when PHs H and P were applied to NaCl-stressed tomato plants, there was a significant decrease in Na^+^ content (by 12% and 25%, respectively) when compared to high salt control plants (**Figure 2A**), yet no remarkable differences were observed for K^+^ concentration (**Figure 2B**).

On the other hand, in control lettuce plants, salt stress induced an increase of 246% in the Na^+^ concentration compared to no salt plants; when considering the application of PHs, the most remarkable effect was produced by P, which reduced Na^+^ concentration by about 21% compared to P-treated non-stressed plants (**Figure 2D**). Considering the data obtained in lettuce plants subjected to high salt stress, differential effects were triggered by the PHs application. In fact, PHs P and D reduced Na^+^ concentration by about 69%, H by 56% and C by 34% compared to salt-stressed control plants (**Figure 2D**). In addition, under high salt conditions, K^+^ concentration decreased by 15% in untreated-control plants and by 11% in P-treated plants (**Figure 2E**), whilst the application of C increased K^+^ concentration by 28%, and no significant differences were observed for D and H, when compared to the same treatments under no-salt conditions (**Figure 2E**).

As a consequence of the changes in both elements induced by the use of different PHs, the Na^+^/K^+^ ratio of salt-stressed tomato plants significantly decreased in tomato plants treated with PHs H and P (by 12% and 23%, respectively) (**Figure 2C**), whereas for salt-stressed lettuce plants all PHs decreased the Na^+^/K^+^ ratio by 73% (D), 65% (P and H) and 45% (C), compared to NaCl-control plants (**Figure 2F**).

### Osmolytes and antioxidative enzyme activities in leaves

**Figure 3:**
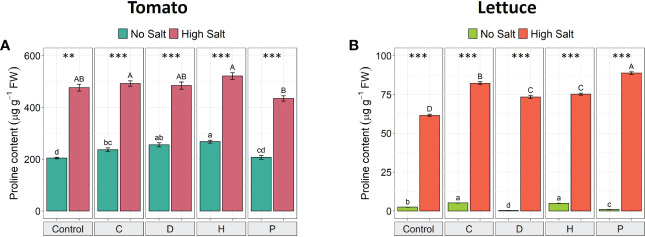
Effect of salinity and protein hydrolysates in the proline accumulation of tomato **(A)** and lettuce **(B)** plants. Values are means ± SE. Lowercase letters compare treatments under no salt, and capital letters compare treatments under high salt. Equal letters correspond to average values that do not differ according to Tukey’s HSD test (p < 0.05). Asterisks indicate significant differences between high and no salt, according to Student’s *t-*test (^**^p < 0.01, ^***^p < 0.001).

The concentration of the osmolyte proline increased in the leaf tissue of both plant species when exposed to salinity stress (**Figure 3**). In salt-stressed tomato plants, proline concentration increased by about 100%, compared to non-stressed plants, independently of the PH applied (**Figure 3A**). On the other hand, in lettuce plants, salt stress induced an increase in the accumulation of proline by about 25-fold in untreated plants. However, the highest proline concentration was detected in salt-stressed lettuce treated with PHs D and P (increased by 200-fold and 90-fold, respectively) (**Figure 3B**). Interestingly, under non-saline conditions, the constitutive accumulation of proline in tomato plants was notably higher than in lettuce plants (by 80-fold). Moreover, the treatment of salt-stressed tomato plants with PHs did not induce significant effects in the accumulation of proline, while under no-salt conditions PHs C, D and H significantly increased this osmolyte compared to the corresponding control plants (**Figure 3A**). In salt-stressed lettuce plants, all PHs induced a significant increase of this osmolyte (ranging from 19-44%) compared to NaCl-control plants, whilst under normal conditions, only PHs C and H enhanced proline content (**Figure 3B**).

High salinity also stimulated the activity of antioxidant enzymes (CAT, APX, GPX and SOD) in leaves of both plant species, but APX and GPX were more enhanced in tomato, whereas CAT and APX were the most induced in lettuce (**Table 2**). In addition, the use of biostimulants promoted differential responses that were dependent on the plant species and the nature of the PHs applied. For instance, in lettuce plants grown under salinity stress, the use of PHs P and H significantly increased the activity of most of the antioxidant enzymes compared to untreated-control plants (**Table 2**), whereas in stressed-tomato plants each PH was efficient in enhancing the activity of a given enzyme.

**Table 2:** Effects of salinity stress and protein hydrolysates application on antioxidant enzyme system in tomato and lettuce plants.

Antioxidant enzymes	Salt levels	Tomato					Lettuce				
		Biostimulant treatment					Biostimulant treatment				
		Control	C	D	H	P	Control	C	D	H	P
CAT	Low salt	5.52 Bb	5.92 Bb	8.86 Ba	6.72 Bb	8.73 Ba	4.28 Be	5.57 Bd	8.49 Bb	14.45 Ba	7.57 Bc
	High salt	18.35 Abc	22.17 Aa	19.77 Aab	16.53 Ac	22.05 Aa	28.52 Ab	31.48 Aab	32.30 Aa	32.69 Aa	32.13 Aa
APX	Low salt	194.67 Bb	216.95 Bab	259.11 Ba	195.55 Bb	252.89 Ba	177.33 B	176.19 B	179.51 B	198.28 B	197.06 B
	High salt	719.09 A	761.05 A	790.78 A	712.90 A	720.04 A	427.01 Ab	338.28 Ad	415.85 Abc	382.55 Ac	523.44 Aa
GPX	Low salt	171.16 Bb	191.76 Bab	211.38 Ba	140.08 Bc	167.69 Bbc	3.74 Bc	3.30 Bcd	2.85 Ba	6.46 Bd	5.26 Bb
	High salt	546.16 Ab	667.07 Aa	699.91 Aa	629.89 Aa	490.64 Ab	13.57 Ad	17.09 Ac	13.16 Ad	30.78 Aa	20.43 Ab
SOD	Low salt	1.31 Bb	1.36 Bb	1.63 Ba	1.05 Bc	1.70 Ba	0.71 Bb	0.64 Bb	0.66 Bb	1.10 Ba	1.13 Ba
	High salt	4.87 Aabc	4.83 Abc	5.20 Aab	5.47 Aa	4.55 Ac	2.60 Ab	2.11 Ac	3.27 Aa	3.43 Aa	3.16 Aa

Differences between biostimulant treatments were determined using Tukey’s HSD test, and significant differences (p < 0.05) are indicated by different lowercase letters when comparing means in rows. Salt level effects were compared using Student’s t-test and significant differences (p < 0.05) are indicated by different capital letters when comparing means in columns. No significant differences are indicated by omitting notation letter.

### *PAL* gene expression in leaves

**Figure 4:**
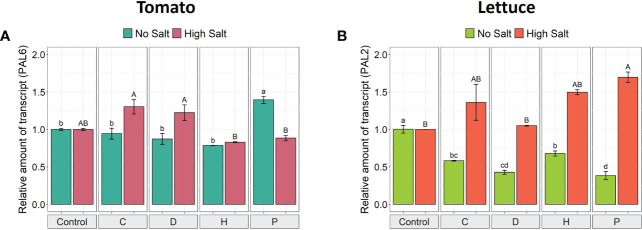
Gene expression analysis of *PAL6* in leaves of tomato **(A)** and *PAL2* in leaves of lettuce **(B)** grown under salinity stress and protein hydrolysates application. The expression levels of *PAL6* and *PAL2* genes were normalized to the expression levels of the elongation factor isoform 1-alpha (EF-1a) and the adenosine phosphoribosyl transferase (APT1), respectively. The relative expression ratios were calculated using the control treatment in each salinity condition as a calibrator sample. Values are means ± SE; n = 3. Lowercase letters compare treatments under no salt, and capital letters compare treatments under high salt. Equal letters correspond to average values that do not differ according to Tukey’s HSD test (p < 0.05).

Since phenylalanine ammonia lyase (PAL; EC 4.3.1.5) is a key upstream enzyme in synthesizing the majority of polyphenolic compounds involved in plant response to stresses (Hoffmann et al., 2021), its transcriptional modulation was studied in order to shed light on its role in plant tolerance to high salinity conditions. The application of PHs influenced the *PAL* gene expression in both plant species subjected to either optimal or high salt conditions (**Figure 4**). In tomato plants grown under high NaCl, PHs C and D induced a slightly higher expression of the *PAL6* gene (1.3-fold) compared to control plants, albeit not significantly. However, under non-saline conditions, the biostimulant P was the only one to induce a higher gene expression (1.4-fold) (**Figure 4A**). On the other hand, the application of P induced significant over-expression of *PAL2* in salt-stressed lettuce plants (1.7-fold), yet C and H also enhanced its expression (by ~1.4-fold), even though not significant when compared to the saline control. Under no salt conditions, all PHs downregulated the *PAL2* expression in lettuce leaves compared to the untreated control (**Figure 4B**).

### PCA of plant responses to PHs application and salt stress

**Figure 5:**
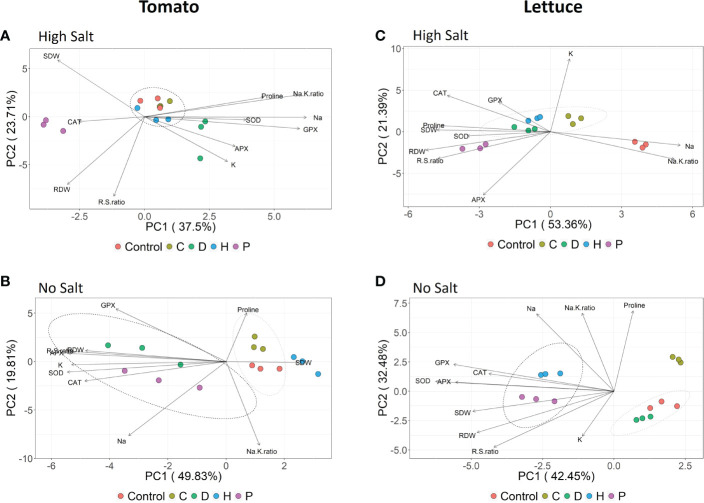
Principal component analysis (PCA) of major traits measured for tomato **(A, B)** and lettuce **(C, D)** summarizing the responses to vegetal-derived protein hydrolysates application (Control, C, D, H and P) under each salinity condition (no salt: 0 mM NaCl or high salt: 120 mM NaCl and 80 mM NaCl for tomato and lettuce, respectively).

In order to better understand the influence of the single parameters recorded on the overall performance of plants subjected to the different treatments, a principal component analysis (PCA) considering both the agronomical and biochemical data was performed for each plant species. PCA confirmed that salt stress was the prevalent factor influencing the behavior of tomato and lettuce plants (**Supplementary Figure S1**). In this sense, to better understand the possible positive effects of PHs application, separated PCA have been carried out for the two plant species, keeping high salt and no salt conditions separated (**Figure 5**).

In tomato plants subjected to high salinity, the scatterplot obtained by combining the two principal components (PC1 and PC2) accounted for about 61% of the total variance and clearly showed the separation of the plants treated with PHs P and D along the PC1, with respect to a cluster formed by all the other samples (**Figure 5A**). For biostimulant P, the main drivers of the separation were the growth parameters and the CAT activity. At the same time, proline, GPX, Na^+^/K^+^ ratio (PC1) and K (PC2) were important to discriminate biostimulant D. Considering tomato grown under no-salt conditions (**Figure 5B**), the two principal components explained together about 70% of the total variance, showing that PHs D and P presented a similar effect on tomato plants mainly discriminated by the antioxidant enzymes, RDW, R/S ratio and K^+^ content along PC1 axis. On the other hand, C and H biostimulants clustered very close to control samples, suggesting a milder effect on plants compared to the other PHs (**Figure 5B**).

In salt-stressed lettuce plants, the PCA produced a model in which the first two principal components, PC1 and PC2, accounted for about 53% and 21% of data variance, respectively (**Figure 5C**). Under this salinity condition, untreated-control plants were clearly separated along the PC1 from the PHs mainly due to Na^+^ concentration and Na^+^/K^+^ ratio, whereas biostimulant P presented the most distinctive effect when compared to the other three PHs, mainly driven by the growth parameters and proline, as well as the antioxidant enzymes CAT and APX. Regarding the lettuce plants grown under no-salt conditions (**Figure 5D**), 75% of the total variance was explained by combining PC1 and PC2. The PHs P and H presented similar effects on lettuce plants under no saline conditions, strongly driven by the growth parameters and all antioxidant enzymes along the PC1 axis.

## Discussion

Results here presented show that salinity stress shrank biomass accumulation of both vegetable species. However, this effect was particularly pronounced in lettuce. From a general point of view, it is well demonstrated that high salt concentrations within the plant tissues compromise the development of roots and leaves in most crops (Robin et al., 2016). Nevertheless, the severity of symptoms induced by salinity stress depends upon many factors, including species, genotype, phenological stage, salt concentration, and time span of plant exposure to the stress (Giordano et al., 2021). It is interesting to note that in this work the use of protein hydrolysates (PHs) induced a differential response in the plant growth according to the vegetable species, the salinity levels, and the origin of the PHs. In this respect, it should be noted that the bioactive potential of PHs in relation to root growth and leaf biomass is often ascribed to the stimulation of cell proliferation associated with the amino acids and peptides composing the PHs, which work as signaling molecules involved in the N metabolism (Caruso et al., 2019). All the four PHs supplied in the present work efficiently enhanced the growth of both root and shoot of salt-stressed lettuce plants, albeit the *Poaceae*-derived PH (P) induced more remarkable effect. On the other hand, none of the four tested PHs induced significant effects on leaves of salt-stressed tomato, whilst only D (Trainer^®^) and P improved root growth. Under abiotic stresses, PHs have been described to trigger several physiological and metabolic mechanisms, including plant hormone regulation, chlorophyll-related metabolism and stress-related metabolism (Lucini et al., 2015; Rouphael et al., 2017; Sorrentino et al., 2022). However, due to the variable composition of PHs, many crop systems respond differently to the biostimulant applied (Parađiković et al., 2019).

Previous studies have demonstrated that salt-tolerant species differ from more sensitive ones in preventing the accumulation of toxic salt levels in leaves (Munns, 2002). In the present work, both crop species showed an elevated Na^+^ ion concentration in leaves, which led to ionic imbalance and decreased the concentration of K^+^ ions. However, this effect was more marked in lettuce plants. Tomato and lettuce, featuring an EC_t_ of 2.5 and 2.0 dS m^−1^, respectively, are considered moderately sensitive to salinity and show adaptive mechanisms to this abiotic stressor (Machado and Serralheiro, 2017). Nonetheless, variation in salt sensitivity is found between species and genotypes, mainly due to the ability to store Na^+^ ions in leaves (Munns et al., 2016). Interestingly, all PHs reduced the concentration of Na^+^ and Na^+^/K^+^ ratio in leaves of lettuce grown under high salinity, whereas only the PHs H and P produced the same effect in tomato. Indeed, maintaining the Na^+^/K^+^ ratio to minimal values in leaves is an important indicator of salinity tolerance (Assaha et al., 2017), clearly suggesting active and differential roles of PHs in modulating ion homeostasis. In this sense, it has been previously reported that applying a plant-based biostimulant to chili pepper plant significantly alleviated the negative effects of salinity stress by rebalancing ions content and modulating phytohormones concentrations (Abou-Sreea et al., 2021).

Under salinity stress, plants can accumulate compatible solutes, such as proline, which play protective roles as an osmoprotectant, scavenging reactive oxygen species (ROS), stabilizing cellular structures and enzymes, and providing cellular redox balance (Meena et al., 2019). Although proline accumulation can be considered a general response to salinity in many plant species, its role in salinity tolerance can be ambiguous and strongly dependent on plant species (Arteaga et al., 2020). In this work, proline content in tomato plants grown under optimal conditions was constitutively higher than lettuce plants. However, under salinity stress, lettuce plants presented a higher accumulation, in terms of fold-change, of this osmolyte over tomato. Depending on the species involved and the severity and duration of the stress, proline content can be accumulated at significantly levels compared to non-stress conditions (Kavi Kishor and Sreenivasulu, 2014). In this context, it has been reported that salt-tolerant species are more efficient in maintaining cell osmolarity under saline conditions, whereas salt-sensitive species need to synthesize higher levels of proline to balance the intracellular osmotic potential (Chen et al., 2007; Kozminska et al., 2018). Therefore, our results suggest that lettuce is more sensitive to salt stress than tomato plants, albeit previous data consider them equally sensitive to salinity (Machado and Serralheiro, 2017). Furthermore, in salt-stressed tomato plants, the foliar application of PHs did not enhance the levels of proline, whereas in salt-stressed lettuce all PHs increased this osmolyte, indicating a correlation between salt-sensitivity and the beneficial effect of PHs. More precisely, the more salt-sensitive is a vegetable species, the higher is the ability of PHs to counteract the adverse effects of salinity.

It is well documented that, to deal with the oxidative damage induced by salinity stress, plants can activate the enzymatic antioxidant defense system represented by enzymes such as catalase (CAT), ascorbate peroxidase (APX), guaiacol peroxidase (GPX), and superoxide dismutase (SOD). In fact, they are all crucial in regulating and/or detoxifying harmful levels of reactive oxygen species (*i.e*., H_2_O_2_, O2^•‾^, HO_2_^•^, RO^•^, ^•^OH) (Hasanuzzaman et al., 2020) as a consequence of the stress. Results here presented show that the activities of the four enzymes were increased under high salinity and showed responses both plant species-specific and PH-related. It has been reported that an enhanced antioxidant defense system induced using biostimulants is directly involved in ROS scavenging and oxidative stress reduction in plants under salinity (Hasanuzzaman et al., 2021). Noteworthy, the specific use of PHs P (*Poaceae*-derived) and H (Vegamin^®^) showed greater potential in eliciting the antioxidant system in lettuce plants, whereas none of the tested PHs could contemporarily upregulate the activities of all the antioxidant enzymes in tomato plants. The fact that a specific PH induces a plant species-specific response in terms of enhanced activity of enzymatic antioxidants may be ascribable, at least in part, to the peptide components in the PH, acting as signal molecules in regulating physiological processes.

Overexpression of specific *PAL* gene isoforms has been reported to improve plant tolerance to several environmental stresses (Olsen et al., 2008; Kim and Hwang, 2014; Zhang et al., 2018). In the present work, when high salinity stress was imposed, some PHs up-regulated the expression of *PAL6* and *PAL2* in tomato and lettuce plants, respectively, but these responses were dependent on the vegetable species and the nature of the biostimulant applied. These results agree with other studies, which have also reported that using plant-based biostimulants can enhance the transcription of a set of stress-related genes, including *PAL* isoforms (Ertani et al., 2011; Ertani et al., 2013; Trevisan et al., 2019). Increased *PAL* activity is generally correlated with the increased production of phenylpropanoids and flavonoids (Vogt, 2010), which are believed to play a key role in plant stress protection by regulating the antioxidant system, photosynthetic system, plasma membrane integrity, and gene expression levels (Yaqoob et al., 2022).

The multivariate statistical analyses further demonstrated the variability observed in the response of the individual vegetable species to biostimulants application under a given salinity condition. All four tested PHs prompted the amelioration of NaCl-induced toxicity in lettuce plants. Yet, the *Poaceae*-derived biostimulant (P) showed the most remarkable effect associated with multiple mechanisms, including enhanced biomass accumulation, improved antioxidant defense machinery, and balanced ionic content. Under non-stress conditions, only P and H (Vegamin^®^) presented promising effects in enhancing lettuce growth and health. On the other hand, applying P and D (Trainer^®^) allowed tomato plants to cope with the adverse effects of salinity through different ways of action: while P stimulated plant growth, D activated the antioxidant system and ion homeostasis. The same two PHs also contributed to plant growth and the general fitness of tomato under no-salt conditions. Indeed, the differential effectiveness of plant-derived PHs can be ascribed to either synergistic or antagonistic effects of several bioactive molecules that are inherently present in the mixtures used (Bulgari et al., 2019). Therefore, the biostimulant properties of protein hydrolysates under normal or saline conditions seem to be strongly correlated to their origin and, thus, their composition, as reported in previous comparative studies using plant-derived biostimulants (Abdel Latef et al., 2017; Abou-Sreea et al., 2021).

Yet, it is very important to further highlight the demonstrated role of PHs in mitigating the harmful effects of abiotic stresses, which are predicted to threaten the agricultural production in the next years. Indeed, the regular application of these natural substances obtained by the valorization of waste biomass could represent on one side a virtuous example of circular economy and, on the other hand, they might constitute an innovative and sustainable agricultural approach.

## Conclusions

The findings provided by this study demonstrate that foliar application of vegetal-derived protein hydrolysates to two different plant species grown under contrasting saline conditions effectively attenuated salinity stress damage to different extents. Our results demonstrated that, albeit being previously assessed as equally sensitive, lettuce plants showed less tolerance to salt stress with respect to tomato plants. In addition, the effectiveness of PHs in counteracting the toxic effects of salinity was more evident in lettuce plants, *i.e*., the most sensitive of the two vegetables used in this study. Nonetheless, we also demonstrated that both the botanical origin and the composition of PHs play a major role in the biostimulants effects on plant growth and stress amelioration. Yet, the *Poaceae*-derived (P) and Trainer^®^ (D) were revealed as the most promising PHs for the amelioration of salt stress in both vegetable species. Overall, the evidence gathered strongly suggests that, to completely exploit the biostimulant potential of PHs in the context of specific abiotic stresses, the correct combination of plant species and PHs needs to be carefully considered. To this purpose, a deeper understanding of the mechanisms underlying the PHs effects on crops represents a fundamental step also for a more focused, efficient, and large-scale use of these natural products in a context of a continuously more sustainable and resilient agriculture.

## Data availability statement

The original contributions presented in the study are included in the article/**Supplementary Material**. Further inquiries can be directed to the corresponding authors.

## Author contributions

MYAZ, SC, and YP conceived the work and designed the experiment. MYAZ and SM carried out the experiments and generated the data. MYAZ and YP analyzed the data. MYAZ wrote the first draft of the manuscript, which was intensively edited by all authors. MYAZ, LL, YR, GC, SC, and YP reviewed the manuscript and carried out the English edition. All authors contributed to the article and approved the submitted version.
