# Supplementary material for: Different vegetal protein hydrolysates distinctively alleviate salinity stress in vegetable crops: A case study on tomato and lettuce
Source: Front Plant Sci. 2023 Feb 15;14:1077140. doi: 10.3389/fpls.2023.1077140 (PMC9975731; doi:10.3389/fpls.2023.1077140)
Supplement: Supplementary file 1 [file DataSheet_1.pdf]

Supplementary Material

Supplementary Table 1. Sequence of forward and reverse primers used in Real-time RT-PCR experiments.

**Plant**	**Gene**	**Forward primer 5-3**	**Reverse primer 5-3**
Tomato	SlEF1α	ACTGTGCAGTGTGATGCTGG	AATACACAACTCAAAGACCAAAC
	SlPAL6	ACTTCAATTATTTTTTACATTTACC	AAGGACTTGTAATAATATATAGCT
Lettuce	LsAPT1	GCTTTGATTGACCAGAGTACT	CATAAACCGAAACAAATCTCATT
	LsPAL2	GTTGTTGGAGTGTCTTGGAG	TATTACAAATACAATTCAAACGGT

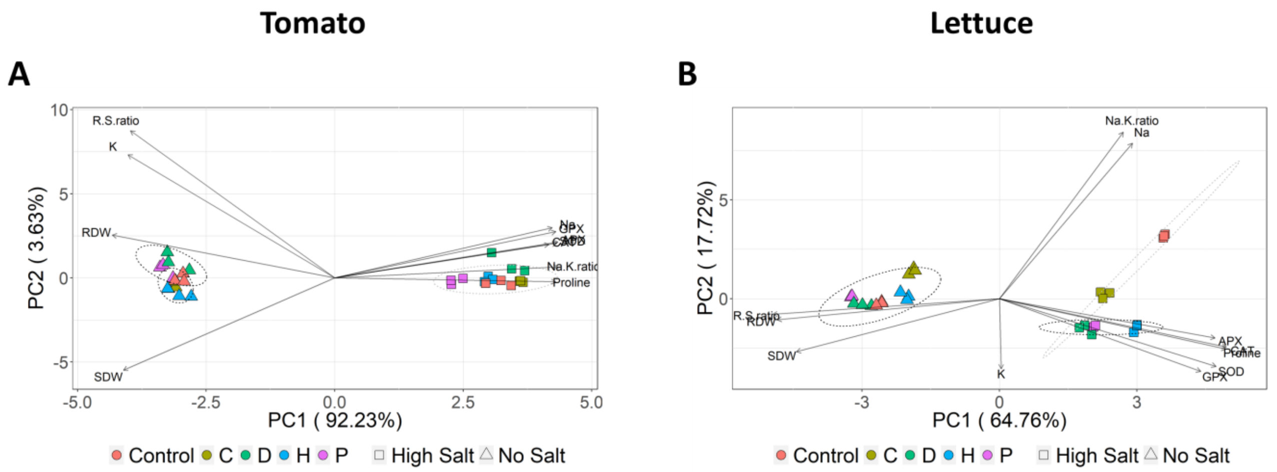

Supplementary Figure 1. Principal component analysis (PCA) of major traits measured for tomato (**A**) and lettuce (**B**) summarizing the responses to vegetal-derived protein hydrolysates application (Control, C, D, H and P) under both salinity conditions (no salt: 0 mM NaCl or high salt: 120 mM NaCl and 80 mM NaCl for tomato and lettuce, respectively).
